# MicroRNA-520b Inhibits Growth of Hepatoma Cells by Targeting MEKK2 and Cyclin D1

**DOI:** 10.1371/journal.pone.0031450

**Published:** 2012-02-03

**Authors:** Weiying Zhang, Guangyao Kong, Junping Zhang, Tao Wang, Lihong Ye, Xiaodong Zhang

**Affiliations:** 1 Department of Cancer Research, Key Laboratory of Molecular Microbiology and Technology of Ministry of Education, Institute for Molecular Biology, College of Life Sciences, Nankai University, Tianjin, People's Republic of China; 2 Department of Biochemistry, College of Life Sciences, State Key Laboratory of Medicinal Chemical Biology, Nankai University, Tianjin, People's Republic of China; Sun Yat-sen University Medical School, China

## Abstract

Growing evidence indicates that the deregulation of microRNAs (miRNAs) contributes to the tumorigenesis. We previously revealed that microRNA-520b (miR-520b) was involved in the complement attack and migration of breast cancer cells. In this report, we show that miR-520b is an important miRNA in the development of hepatocellular carcinoma (HCC). Our data showed that the expression levels of miR-520b were significantly reduced in clinical HCC tissues and hepatoma cell lines. We observed that the introduction of miR-520b dramatically suppressed the growth of hepatoma cells by colony formation assays, 5-ethynyl-2-deoxyuridine (EdU) incorporation assays and 3-(4,5- dimethylthiazol-2-yl)-2,5-diphenyltetrazolium bromide (MTT) assays. Moreover, ectopic expression of miR-520b was able to inhibit the growth of hepatoma cells in nude mice. Further studies revealed that the mitogen-activated protein kinase kinase kinase 2 (MEKK2) and cyclin D1 were two of direct target genes of miR-520b. Silencing of MEKK2 or cyclin D1 was able to inhibit the growth of hepatoma cells *in vitro* and *in vivo*, which is consistent with the effect of miR-520b overexpression on the growth of hepatoma cells. In addition, miR-520b significantly decreased the phosphorylation levels of c-Jun N-terminal kinase (p-JNK, a downstream effector of MEKK2) or retinoblastoma (p-Rb, a downstream effector of cyclin D1). In conclusion, miR-520b is able to inhibit the growth of hepatoma cells by targeting MEKK2 or cyclin D1 *in vitro* and *in vivo*. Our findings provide new insights into the role of miR-520b in the development of HCC, and implicate the potential application of miR-520b in cancer therapy.

## Introduction

MicroRNAs (miRNAs) belong to a class of small, highly conserved noncoding RNAs known to suppress the expression of protein-coding genes through imperfect complementarity with the 3′-untranslated region (3′UTR) of target messenger RNA (mRNA) [1]. Accumulating evidence has shown that miRNAs can function as oncogenes or tumor suppressors involved in cancer development, including tumor metastasis and proliferation [2], [3], [4]. Many studies demonstrate that miRNAs have essential roles in hepatocellular carcinoma (HCC) progression and directly contribute to cell proliferation and metastasis of HCC by targeting a large number of critical protein-coding genes. For example, overexpression of miR-26a induces HCC cell cycle arrest. Cyclin D2 and cyclin E2 are validated as direct targets of miR-26a, which exhibits reduced expression in HCC [5]. MiR-21, miR-221 and miR-222 contribute to the proliferation and metastasis of HCC cells by targeting phosphatase and tensin homolog (PTEN) [6], [7]. MiR-122 is significantly downregulated in liver cancer and suppresses HCC intrahepatic metastasis by regulation of a disintegrin and metalloprotease family proteins ADAM10 and ADAM17 [8], [9]. Although a large number of miRNAs have been identified, their roles in HCC development and the underlying mechanisms still need to be explored. We have reported that miR-520b is downregulated in breast cancer cells relative to normal breast cells, and sensitizes breast cancer cells to complement attack via directly targeting 3′UTR of CD46 [10]. Recently, research in our laboratory has revealed that miR-520b inhibits the migration of breast cancer cells by targeting interleukin-8 (IL-8) and hepatitis B X-interacting protein (HBXIP) [11]. However, the roles of miR-520b in the growth of hepatoma cells remain unclear.

Cyclin D1 is a cell cycle regulator. Aberrant expression of cyclin D1 can lead to abnormal cellular proliferation [12]. Mitogen-activated protein kinase kinase kinase 2 (MEKK2), a member of the MAPK signaling pathway, is able to activate c-Jun N-terminal kinase (JNK) and ERK5 [13], [14]. MEKK2 knockout mouse studies have revealed that MEKK2 has important functions in the T-cell receptor, epidermal growth factor (EGF) and fibroblast growth factor 2 (FGF-2) signaling pathways [13], [15], [16]. It has been reported that MEKK2 is able to discriminate tumor from normal cells [17], suggesting that MEKK2 may play important roles in the development of cancer.

In the present study, we showed that miR-520b was downregulated in HCC tissues and hepatoma cell lines. Our finding shows that miR-520b is able to inhibit the growth of hepatoma cells by targeting MEKK2 and cyclin D1 *in vitro* and *in vivo*. Our data provide new insights into the role of miR-520b in the development of HCC.

## Results

### MiR-520b is downregulated in HCC tissues and hepatoma cell lines

**Figure 1:**
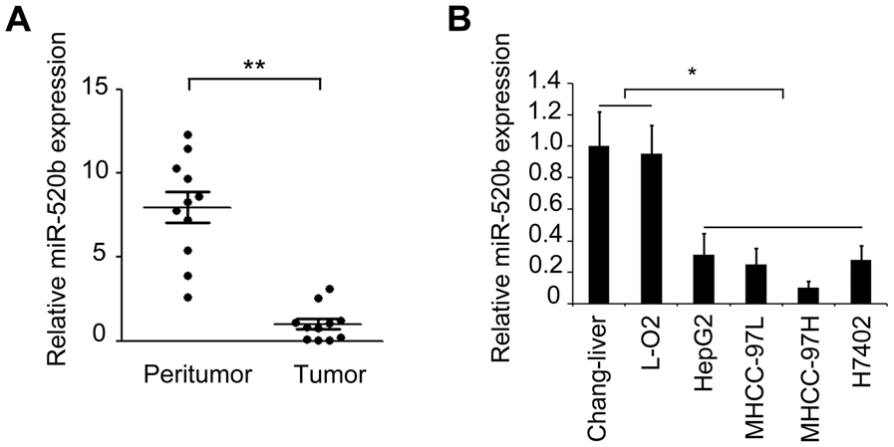
MiR-520b is downregulated in HCC tissues and hepatoma cell lines. (A) The relative expression of miR-520b in clinical HCC patients was examined by qRT-PCR. (B) The relative expression of miR-520b in hepatoma cells and normal liver cells was examined by qRT-PCR. *P<0.05, **P<0.01, Student's *t* test.

Previous study in our laboratory showed that the expression level of miR-520b was downregulated in breast cancer cells compared with normal breast cells [10]. To further investigate the roles of miR-520b in the development of HCC, we analyzed the expression level of miR-520b in 11 paired clinical HCC tissues and adjacent noncancerous liver tissues by quantitative reverse-transcription PCR (qRT-PCR). Clinical characteristics of the patients are listed in Table S1. Compared with their peritumor tissues, significant downregulation of miR-520b was observed in clinical HCC tumor tissues (Fig. 1A, **P<0.01, Student's *t* test). Meanwhile, the expression levels of miR-520b were reduced in 4 hepatoma cell lines relative to 2 normal liver cell lines (Fig. 1B, *P<0.05, Student's *t* test). The data suggest that the expression level of miR-520b is downregulated in human HCC.

### MiR-520b inhibits growth of heptoma cells *in vitro*

**Figure 2:**
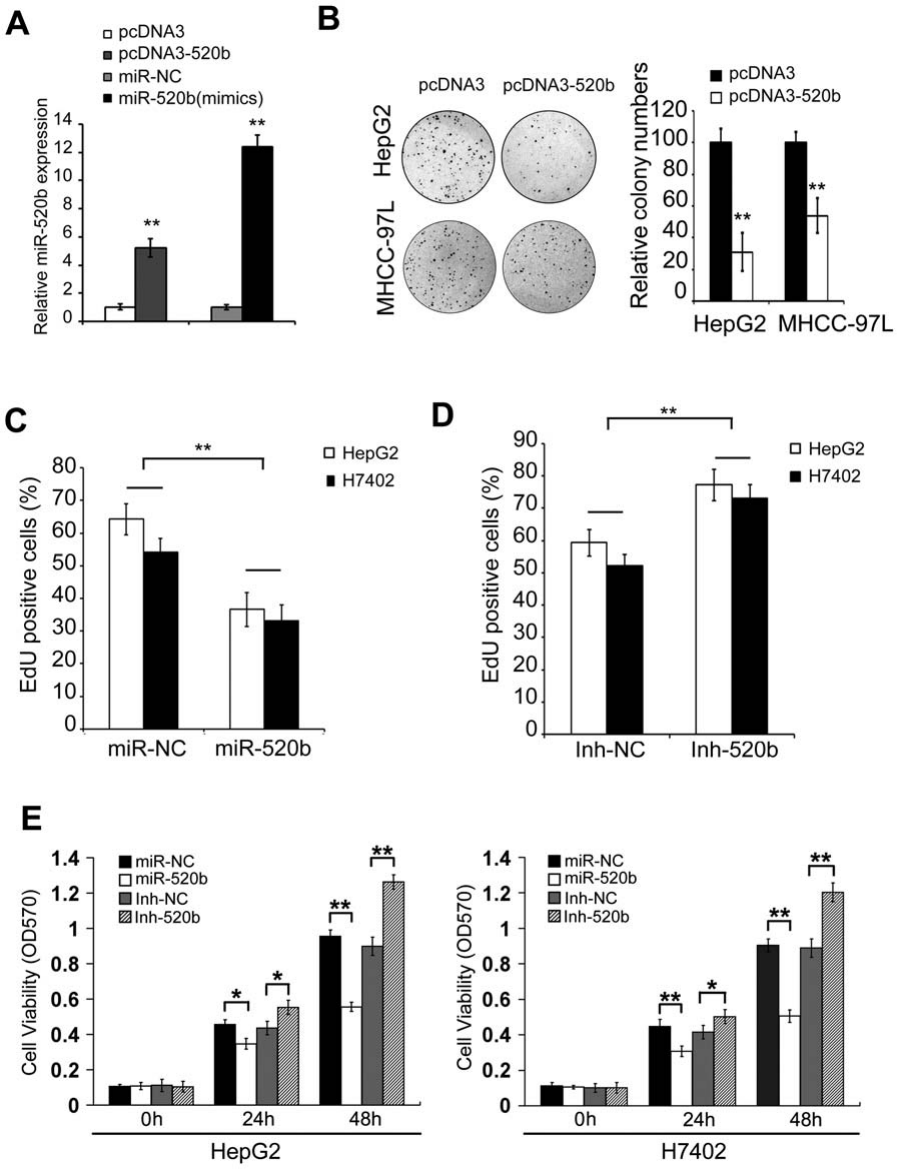
MiR-520b inhibits growth of hepatoma cells *in vitro*. (A) The expression levels of miR-520b were tested by qRT-PCR in HepG2 cells transfected with pcDNA3, pcDNA3-520b, 100 nM miR-520b negative control (termed miR-NC) and 100 nM miR-520b mimics (termed miR-520b). (B) The effect of pcDNA3-520b or pcDNA3 on the growth of HepG2 and MHCC-97L cells was examined by colony formation assay. Data are representative of three independent experiments. (C) The effect of transient transfection of miR-520b or miR-NC on the growth of HepG2 and H7402 cells was examined by EdU incorporation assay. (D) The effect of transient transfection of miR-520b inhibitor (termed Inh-520b) or miR-520b inhibitor negative control (termed Inh-NC) on the growth of HepG2 and H7402 cells was examined by EdU incorporation assay. (E) HepG2 and H7402 cells were transfected with 100 nM miR-NC, 100 nM miR-520b, 200 nM Inh-NC and 200 nM Inh-520b, respectively. The effects of miRNAs on cell proliferation were determined by MTT assay at 0h, 24h and 48h after transfection. *P<0.05, ** *P*<0.01, Student's *t* test.

We demonstrated that the expression level of miR-520b in pcDNA3-520b transfected HepG2 cells was higher than that in pcDNA3 transfected cells by qRT-PCR (Fig. 2A, **P<0.01, Student's *t* test). A more than ten-fold increase in the expression of miR-520b was observed in HepG2 cells transfected with 100 nM miR-520b mimics (termed miR-520b) relative to the cells transfected with 100 nM miR-520b negative control (termed miR-NC) (Fig. 2A, **P<0.01, Student's *t* test). Colony formation analyses indicated that the growth ability of pcDNA3-520b transfected cells was lower than that of pcDNA3 transfected cells (Fig. 2B, **P<0.01, Student's *t* test). The 5-ethynyl-2-deoxyuridine (EdU) incorporation assays showed that the growth of HepG2 cells and H7402 cells was significantly inhibited by 100 nM miR-520b relative to 100 nM miR-NC (Fig. 2C, **P<0.01, Student's *t* test). Meanwhile, miR-520b inhibitor (termed Inh-520b) enhanced the growth of HepG2 cells and H7402 cells compared with the inhibitor negative control (termed Inh-NC) (Fig. 2D, **P<0.01, Student's *t* test). Next, 3-(4,5-dimethylthiazol-2-yl)-2,5- diphenyltetrazolium bromide (MTT) assays demonstrated that the transient overexpression of miR-520b resulted in the inhibition of the growth of HepG2 and H7402 cells; while Inh-520b significantly attenuated the suppression (Fig. 2E, *P<0.05, **P<0.01, Student's *t* test). Thus, our data indicate that miR-520b is able to inhibit the growth of hepatoma cells *in vitro*.

### MiR-520b suppresses tumorigenicity of hepatoma cells *in vivo*

**Figure 3:**
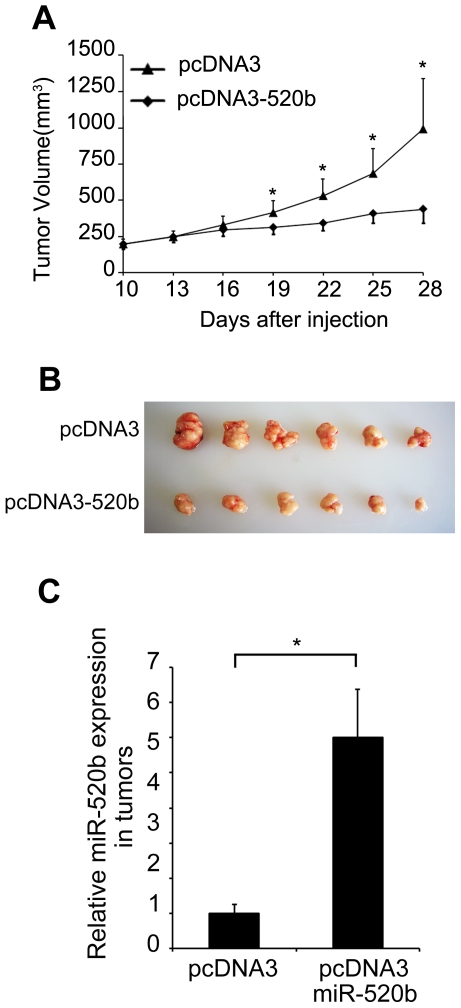
MiR-520b inhibits growth of hepatoma cells *in vivo*. (A) The curve of tumor growth is shown. (B) Photograph of excised tumors 4 weeks after implantation. (C) qRT-PCR analysis of miR-520b expression in excised tumors was performed and normalized against an endogenous control (U6 RNA).

We further investigated the function of miR-520b *in vivo* using xenograft model. Stably pcDNA3-520b-tranfected HepG2 cells were injected subcutaneously into 6 female BALB/c athymic nude mice, respectively. After 4 weeks, we found that introduction of pcDNA3-520b into HepG2 cells led to a significant reduction of tumor volume. qRT-PCR analyses demonstrated that the expression levels of miR-520b were obviously increased in pcDNA3-520b tumors compared with control tumors (Fig. 3A, B and C, *P<0.05, Student's *t* test). The data provide evidence that miR-520b can inhibit the growth of hepatoma cells *in vivo*.

### MiR-520b directly inhibits expression of MEKK2 and cyclin D1 via their 3′UTR

**Figure 4:**
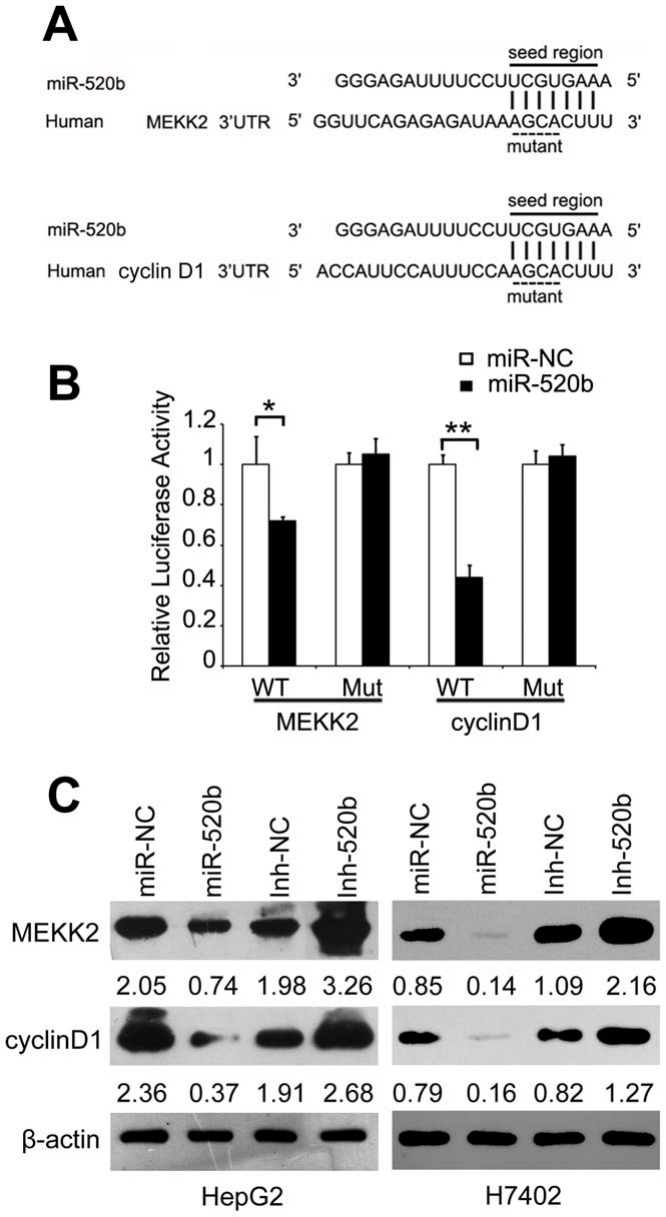
MiR-520b directly inhibits expression of MEKK2 and cyclin D1 *via* their 3′UTR. (A) Sequence alignment between miR-520b and the 3′UTR of human MEKK2 and cyclin D1 mRNA. Solid line, seed match region; dashed line, seed-mutated region. (B) The effect of miR-520b on the activity of firefly luciferase reporter containing either wild type (WT) or mutant type (Mut) 3′UTR was tested by luciferase reporter gene assays. (C) The effect of miR-520b or Inh-520b on the endogenous expression levels of MEKK2 and cyclin D1 was examined in HepG2 and H7402 cells by western blot analyses. β-actin was used as an internal control. The intensity for each band was quantified densitometrically.

To explore the mechanisms of miR-520b-induced cell growth inhibition, we searched for the target genes of miR-520b. The regions complementary to miR-520b seed region in the 3′UTR of human MEKK2 or cyclin D1 mRNA were observed by using DIANA microT v3.0 algorithm (Fig. 4A), which was also confirmed by TargetScan and PicTar. To validate whether MEKK2 and cyclin D1 were the direct target genes of miR-520b, a dual-luciferase reporter system was employed. We cloned 3′UTR sequences containing the predicted target site (wild type, WT) of miR-520b or mutated sequences (mutant type, Mut) into the pGL3 control vector, respectively. The data showed that the co-expression of miR-520b mimics significantly suppressed the firefly luciferase activities of the reporter with wild type 3′UTR but not that of the mutant reporter (Fig. 4B, *P<0.05, **P<0.01, Student's *t* test), indicating that miR-520b can directly target the 3′UTR of MEKK2 and cyclin D1. The effect of miR-520b on endogenous expression of MEKK2 and cyclin D1 was subsequently examined by western blot. Transfection of miR-520b resulted in an obvious downregulation of MEKK2 and cyclin D1 at the protein levels in HepG2 and H7402 cells. Meanwhile, miR-520b inhibitor was able to upregulate the expression of MEKK2 and cyclin D1 in the cells (Fig. 4C). Western blot analyses showed that the expression levels of MEKK2 and cyclin D1 were decreased dramatically in 520b-overexpressed tumors from mice (Fig. 3) relative to control tumors (Fig. S1). It suggests that miR-520b is able to downregulate the expression of MEKK2 and cyclin D1 in the tumor cells.

### MiR-520b targeted MEKK2 and cyclin D1 contribute to growth of hepatoma cell *in vitro* and *in vivo*

**Figure 5:**
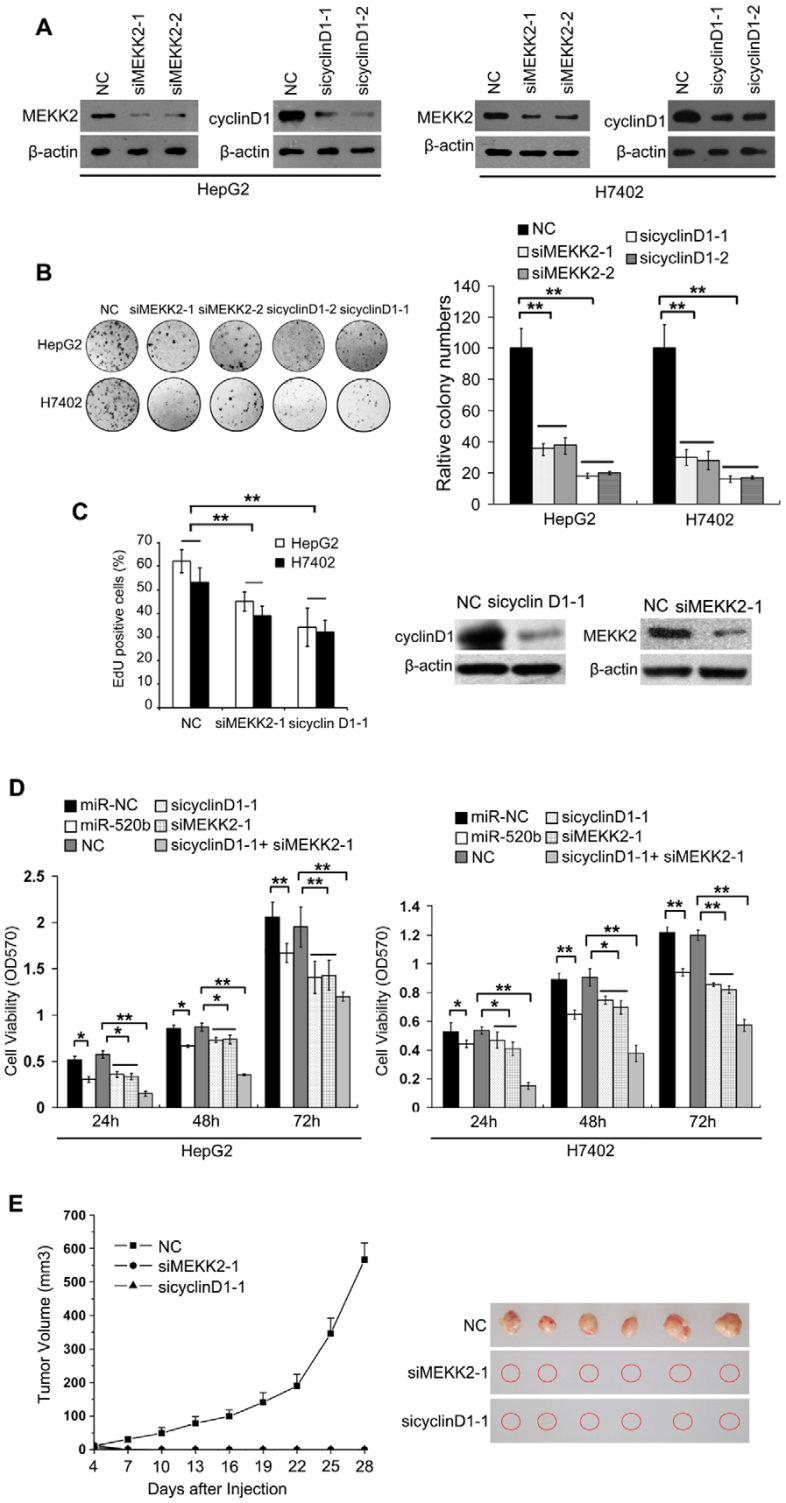
MiR-520b targeted MEKK2 and cyclin D1 contribute to hepatoma cell growth *in vitro* and *in vivo*. (A) The expression levels of MEKK2 and cyclin D1 were examined by western blot analyses in HepG2 and H7402 cells treated with two different siRNAs targeting MEKK2 (termed siMEKK2-1 and siMEKK2-2) or two different siRNAs targeting cyclin D1 (termed sicyclinD1-1 and sicyclinD1-2). (B) The effect of transient transfection of siRNAs targeting MEKK2 (siMEKK2-1 or siMEKK2-2) or cyclin D1 (sicyclin D1-1 or sicyclin D1-2) on the growth of HepG2 and H7402 cells was examined by colony formation assays. (C) The effect of transient transfection of sicyclin D1-1 or siMEKK2-1 on the growth of HepG2 and H7402 cells was examined by EdU incorporation assays. (D) HepG2 and H7402 cells were transfected with miR-NC, miR-520b, NC, sicyclin D1-1 and/or siMEKK2-1, respectively. The effects of miRNAs or siRNA targeting cyclin D1 or MEKK2 on hepatoma cell proliferation were determined by MTT assays at 24 h, 48 h and 72 h after transfection. *P<0.05, **P<0.01, Student's *t* test. (E) Tumor growth measured after subcutaneous injection of HepG2 cells transient transfected with NC, siMEKK2-1 or sicyclinD1-1. The tumor volume was calculated every 3 days. Points, mean (n = 6); bars, SD.

To explore whether miR-520b targeted MEKK2 or cyclin D1 is responsible for the inhibition of the growth of hepatoma cells, we examined the effects of knockdown of target genes on the growth of hepatoma cells. Western blot analyses demonstrated that both of two siRNAs targeting MEKK2 (or cyclin D1) were able to effectively knockdown the expression of MEKK2 (or cyclin D1) in HepG2 cells and H7402 cells (Fig. 5A). Colony formation assays showed that knockdown of MEKK2 (or cyclin D1) was able to significantly decrease the colony formation capability in HepG2 (or H7402) cells transfected with two siRNAs targeting MEKK2 (or cyclin D1) relative to control (termed NC) (Fig. 5B, **P<0.01, Student's *t* test). Then, we chose one of siRNAs (termed siMEKK2-1 or sicyclinD1-1) targeting MEKK2 or cyclin D1 to perform EdU and MTT assays, respectively. EdU assays showed that the silencing of the genes led to a significant reduction of growth of HepG2 cells and H7402 cells (Fig. 5C, **P<0.01, Student's *t* test). The silencing efficiency MEKK2 and cyclin D1 were showed by western blot analyses in the experiment (Fig. 5C). MTT assays showed that the transfection of miR-520b or silencing the two target genes (or one gene) was able to dramatically decrease the growth of hepatoma cells relative to controls, miR-NC or NC-transfected cells (Fig. 5D, *P<0.05, **P<0.01, Student's *t* test). To further determine the effect of miR-520b targeted MEKK2 or cyclin D1 on growth of hepatoma cells *in vivo*, the HepG2 cells transiently transfected with siRNAs targeting MEKK2 or cyclin D1 were subcutaneously injected into 4- to 6-week-old BALB/c athymic nude mice. Four days after injection, we found that three group mice (NC, siMEKK2 and sicyclinD1) all formed tumors and the average tumor volumes from the groups of NC, siMEKK2 and sicyclinD1 were 11.45 mm^3^, 6.06 mm^3^ and 12.68 mm^3^, respectively. Seven days later, NC group continually grew. But the tumors of siMEKK2 and sicyclinD1 groups stop to grow, we did not find any tumors 4 weeks latter (Fig. 5E), suggesting that MEKK2 and cyclin D1 play very important roles in hepatocarcinogenesis. Taken together, our finding suggests that miR-520b targeted MEKK2 and cyclin D1 are involved in the tumorigenicity of hepatoma cells in v*itro* and *in vivo*.

### Cyclin D1 overexpression rescues miR-520b depressed growth of hepatoma cells

**Figure 6:**
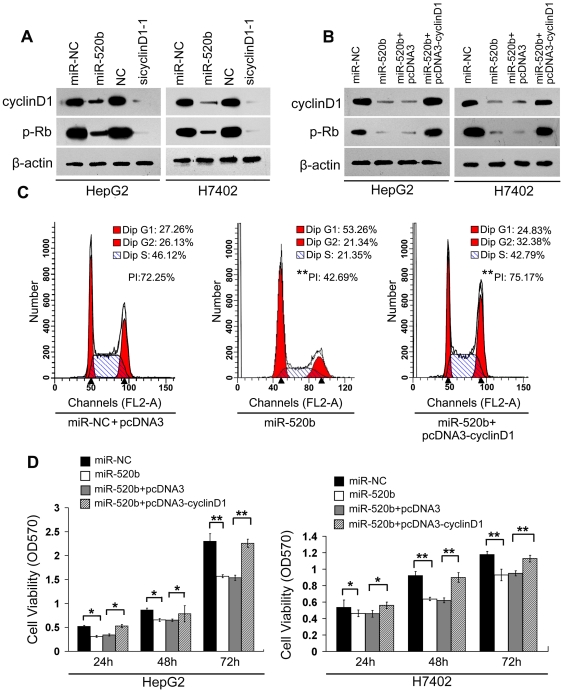
Cyclin D1 overexpression rescues miR-520b depressed growth of hepatoma cells. (A) The effect of miR-520b or sicyclinD1-1 on phosphorylation levels Rb (p-Rb) was examined in HepG2 and H7402 cells by western blot analyses. (B) Western blot analyses showed the expression levels of cyclin D1 and p-Rb in HepG2 and H7402 cells treated by miR-520b or both pcDNA3-cyclin D1 and miR-520b. (C) The effect of cyclin D1 overexpression on the miR-520b-inhibited proliferation of HepG2 cells was examined by flow cytometry analyses. (D) The effect of cyclin D1 overexpression on the miR-520b-inhibited proliferation of HepG2 cells was examined by MTT assays at 24 h, 48 h and 72 h after transfection. *P<0.05, ** *P*<0.01, Student's *t* test.

It has been reported that JNK is one of downstream effectors of MEKK2 [13]. Retinoblastoma (Rb) phosphorylation and cellular growth are promoted by the activation of cyclin D1 in numerous cancers [18]. We further examined the effect of miR-520b on regulation of MEKK2 and cyclin D1 through determining the phosphorylation levels of JNK (a downstream effector of MEKK2) and Rb (a downstream effector of cyclin D1). We found that the transfection with miR-520b in hepatoma cells led to the downregulation of cyclin D1 and decreased the levels of p-Rb (Fig. 6A). Meanwhile, we tested the expression of MEKK2 and p-JNK in the cells as well (Fig. S2). The silencing of cyclin D1 (or MEKK2) could efficiently downregualte the expression of cyclin D1 and decrease the phosphorylation levels of Rb (or JNK) in the cells as above. Interestingly, the overexpression of cyclin D1 was able to rescue the miR-520b-decreased levels of p-Rb by transient transfection with pcDNA3-cyclinD1 plasmids in HepG2 and H7402 cells (Fig. 6B). These data support that MEKK2 and cyclin D1 are two of targeting genes of miR-520b. Next, flow cytometry analyses showed that the transient transfection with miR-520b led to a decreased cell proliferation index (PI) from 72.25% to 42.69% in HepG2 cells (**P<0.01, Student's *t* test). However, the introduction of cyclin D1 resulted in an increased cell PI (from 42.69% to 75.17%, **P<0.01, Student's *t* test) and decreased the percentage of cells in the G1 phase (from 53.26% to 24.83%) after co-transfection with miR-520b and pcDNA3-cyclin D1 plasmids (Fig. 6C). MTT assays showed that the enforced expression of miR-520b dramatically inhibited the proliferation of hepatoma cells and the overexpression of cyclin D1 was able to rescue miR-520b-inhibited proliferation of HepG2 and H7402 cells (Fig. 6D). Thus, our data suggest that cyclin D1 overexpression rescues the inhibition of hepatoma cell growth mediated by miR-520b.

## Discussion

Ectopic expression of miRNAs has been observed in various types of cancers [19], [20], but the current knowledge about miRNAs function in HCC is still preliminary. Therefore, identifying the miRNAs and their targets that are essential for HCC progression may provide promising therapeutic opportunities. Our laboratory previously found that the expression level of miR-520b was significantly downregulated in breast cancer cells. miR-520b inhibits the migration of breast cancer cells [11]. However, the mechanism of miR-520b in the development of cancer remains unclear. In this study, we focused on the investigation of roles of miR-520b in growth of hepatoma cells through its target genes.

We showed that miR-520b was downregulated in both HCC tissues and hepatoma cell lines, suggesting that miR-520b might be implicated in tumorigenesis (Fig. 1). Thus, we are interested in the effect of miR-520b on tumor cell growth. Interestingly, the ectopic expression of miR-520b inhibited the growth of hepatoma cells *in vitro* and *in vivo* (Fig. 2 and 3), suggesting that the downregulation of miR-520b may be responsible for the enhanced growth of hepatoma cells and subsequently facilitate the development of HCC. To explore the mechanism underlying the suppression of hepatoma cell growth mediated by miR-520b, we next set out to identify the target genes of miR-520b. According to bioinformatics analysis, miR-520b may target many genes. Then, we chose two of target genes of miR-520b, such as MEKK2 and cyclin D1 which play important roles in the cell cycle and the cell growth, to demonstrate the mechanism of miR-520b in regulation of the growth of hepatoma cells. Our data show that miR-520b was able to directly target the 3′UTR of MEKK2 and cyclin D1. Western blot analyses showed that the overexpression of miR-520b was able to downregulate the expression of MEKK2 and cyclin D1 at protein levels in HepG2 and H7402 cells (Fig. 4). Cyclin D1 is one of the most important proteins regulating the cell cycle, and related with the development of many cancers. The role of cyclin D1 in cell proliferation is well demonstrated [12], [21]. It leads to G1 cell cycle arrest and inhibits cell growth. MAPKs are very important signaling components that convert extracellular stimuli into cell proliferation. MEKK2 is a member of the MAPK signaling pathway. Our data showed that silencing either MEKK2 or cyclin D1 was able to inhibit the growth of hepatoma cells *in vitro*. Furthermore, tumor formation assays demonstrated that the introduction of siMEKK2 or sicyclin D1 completely inhibited the tumor growth *in vivo* (Fig. 5). These data suggest that MEKK2 or cyclin D1 plays crucial roles in hepatocarcinogenesis. MEKK2 and cyclin D1 may serve as therapeutic targets of liver cancer.

MEKK2 is able to activate JNK. Subsequently, JNK regulates multiple cellular functions including carcinogenesis [22]. Cyclin D1 is involved in directly regulating Rb phosphorylation and cell cycle progression. Hyperphosphorylation of Rb promotes cell cycle progression and cell growth [23], [24]. Here, our data showed that miR-520b was able to downregulate the expression of cyclin D1 or MEKK2 and decrease the levels of p-Rb (a downstream effector of cyclin D1) or p-JNK (a downstream effector of MEKK2), supporting that cyclin D1 and MEKK2 are two of target genes of miR-520b. Furthermore, overexpression of cyclin D1 was able to rescue the miR-520b-decreased levels of p-Rb and proliferation of HepG2 and H7402 cells (Fig. 6 and S2). Thus, we conclude that miR-520b inhibits the growth of hepatoma cells through targeting MEKK2 and cyclin D1. Our findings provide new insights into the role of miR-520b in the development of HCC.

In summary, in this study we report that miR-520b is downregualted in clinical hepatoma tissues and hepatoma cell lines. MEKK2 and cyclin D1 are two of direct targets of miR-520b. MiR-520b is able to inhibit the growth of hepatoma cells through the target genes. Our findings implicate the potential application of miR-520b as a tumor suppressor in cancer therapy.

## Materials and Methods

### Ethics statement

All HCC samples were obtained from patients who signed informed consent approving the use of their tissues for research purposes after operation. The study was approved by the Institutional Review Board at the Nankai University and the Institutional Review Board at Tianjin First Center Hospital. All mouse experiments were approved by the Institutional Animal Care and Use Committee at College of Life Sciences at Nankai University (Approval ID 201009080081). Experimental procedures were performed in accordance with the Guide for the Care and Use of Laboratory Animals (NIH Publication No. 80-23, revised 1996).

### Patient samples

The 11 clinical HCC tissues and the corresponding nearby noncancerous livers used in this study were obtained from patients who underwent radical resection at Tianjin First Center Hospital (Tianjin, China). Specimens were immediately frozen and stored at −80°C. The relevant characteristics of the studied subjects were shown in Table S1. Informed consent was obtained from each patient and the study was approved by the Institute Research Ethics Committee at Nankai University.

### RNA extraction and quantitative reverse transcription PCR (qRT-PCR)

All RNA was extracted from cells or tissues with Trizol reagent according to the manufacturer's instructions (Invitrogen, Carlsbad, CA). To quantify mature miR-520b expression, total RNA was polyadenylated by poly (A) polymerase (Ambion, Austin, TX) according to the manufacturer's protocol. After reverse transcription, qRT-PCR was performed using the quantitative SYBR Green PCR kit (TaKaRa Bio, China). One primer is miRNA-specific, and the other is a universal primer. U6 small nuclear RNA was used as an internal normalized reference. To examine the specificity of the qRT-PCR, the dissociation curve analysis was performed after a completed PCR. All primers used are listed in Table S2.

### Construction of plasmids

To construct a plasmid expressing miR-520b, we amplified the fragment containing miR-520b precursor from HepG2 genomic DNA. The amplified fragment was cloned into a pcDNA3 vector, which was termed pcDNA3-520b. The amplified full-length cyclin D1 from HepG2 cDNA was cloned into a pcDNA3 vector, termed pcDNA3-cyclin D1. An empty pcDNA3 vector was used as control. To construct 3′UTR reporter plasmids, the 3′UTR fragments of human MEKK2 and cyclin D1 mRNA containing the putative miR-520b binding site were amplified from HepG2 cDNA, and cloned into the region downstream of the firefly luciferase gene in the pGL3 control vector (Promega, USA). To introduce four-point-mutation into the seed region of the miR-520b binding site, we used a PCR approach where the seeds sequences were mutated in the primers. All primers used are listed in Table S2.

### Cell culture

Human HCC H7402 cell line, human immortalized liver L-O2 and Chang-liver cell lines were cultured in RPMI Medium 1640 (GIBCO, Grand Island, NY) containing 100 U/ml penicillin, 100 µg/ml streptomycin and 10% fetal calf serum (FCS, Life Technologies, Carlsbad, CA). Human hepatoma HepG2 cell line, human HCC MHCC-97H and MHCC-97L cell lines were cultured in Dulbecco's Modified Eagle's medium (DMEM, GIBCO-BRL, Grand Island, NY) containing 100 U/ml penicillin, 100 µg/ml streptomycin and 10% FCS supplemented with 10% fetal bovine serum. To generate stably pcDNA3-520b- or pcDNA3-transfected HepG2 cell lines, the transfection was performed using Lipofectamine 2000 (Invitrogen). Forty-eight hours after transfection, the transfected cells were incubated in selection medium containing 800 µg/ml G418 (Genview, IL, USA), continuously for 3–4 weeks. The successful stable transfection of pcDNA3-520b into HepG2 cells was confirmed by qRT-PCR.

### MiRNA and RNA interference

MiR-520b mimics (miR-520b), miR-520b negative control (miR-NC), miR-520b inhibitor (Inh-520b), miR-520b inhibitor negative control (Inh-NC), siRNAs duplexes targeting human MEKK2 (siMEKK2-1 or siMEKK2-2) and cyclin D1 (sicyclin D1-1 or sicyclin D1-2) [25], [26] were synthesized and purified by RiboBio (Guangzhou, China). SiRNA duplexes with non-specific sequences were used as siRNA negative control (NC). RNA oligonucleotides were transfected by using Lipofectamine RNAiMAX (Invitrogen) and medium was replaced 6 hours after transfection. A final concentration of 100 nM miR-520b, 200 nM Inh-520b, 100 nM siMEKK2 or 100 nM sicyclin D1 was used unless indicated. RNA transfection efficiency is approximately 70%–80% and the overexpression of miRNA or siRNA persists for at least 48 hours. Lipofectamine 2000 (Invitrogen) was used for transfection of plasmid alone or together with RNA oligonucleotides. All oligonucleotide sequences are listed in Table S2.

### Colony formation assay

HepG2, H7402 and MHCC-97L cells were seeded in 6-well plates. Cells were transfected with plasmids of pcDNA3, pcDNA3-520b or siRNAs targeting MEKK2 or cyclin D1. Forty-eight hours after transfection of pcDNA3-520b, the medium was replaced with fresh medium containing G418 to kill untransfected cells. Cells were grown for 9 to 14 days with fresh media. G418 was added every 3 days in pcDNA3- or pcDNA3-520b-transfected cells. Once colonies were visible, they were stained with methylene blue and photographed. All experiments were performed at least three times. The expression levels of miR-520b, MEKK2 or cyclin D1 in transfected cells were confirmed by qRT-PCR or western blot anlysis.

### Analysis of cell proliferation

Cell proliferation was determined by MTT (Sigma, Aaint Louis, MI) assay as described previously [27] and EdU incorporation assay was carried out using the Cell-Light TM EdU imaging detecting kit according to the manufacturer's instructions (RiboBio). EdU is a thymidine analog whose incorporation can be used to label cells undergoing DNA replication [28].

### Flow cytometry analysis

After 48 hours transfection as earlier described, the cells (1×10^6^) were harvested and washed twice with PBS. Washed cells were resuspended in 0.6 mL PBS, and fixed by the addition of 1.4 mL 100% ethanol at 4°C overnight. The fixed cells were rinsed twice with PBS, and resuspended in propidium iodine (PI) solution, including 50 µg/mL PI and 50 µg/mL RNaseA (Sigma) in PBS without calcium and magnesium, and incubated at 37°C for 30 minutes in the dark. Stained cells were passed through a nylon-mesh sieve to remove cell clumps and analyzed by a FACScan flow cytometer and Cell Quest analysis software (Becton Dickinson, San Jose, CA, USA). Flow cytometry analysis was repeated 3 times.

### Dual-Luciferase reporter gene assay

Luciferase reporter gene assay was performed using the Dual-Luciferase Reporter Assay System (Promega) according to the manufacturer's instructions. Cells of 90% confluence were seeded in 24-well plates. For MEKK2 3′UTR and cyclin D1 3′UTR luciferase reporter assay, wild type or mutant reporter constructs (termed WT or Mut) were co-transfected into HepG2 cells in 24-well plates with 100 nM miR-520b or 100 nM miR-NC and Renilla plasmid by using lipofectamine 2000 (Invitrogen). Reporter gene assays were performed 48 hours post-transfection using the Dual luciferase assay system (Promega, Madison, WI). Firefly luciferase activity was normalized for transfection efficiency using the corresponding Renilla luciferase activity. All experiments were performed at least three times.

### Western blot analysis

Cells were washed in phosphate-buffered saline (PBS), and cellular proteins were extracted in RIPA buffer (Biomed, China). Lysates were cleared by centrifugation, and proteins were separated by gel electrophoresis. Membranes were blocked in PBS-0.1% Tween20 (PBS-T)/5% (w/v) milk for 1 hour at room temperature. Membranes were then incubated with primary antibodies diluted in PBS-T for 2 hours at room temperature. Subsequently, membranes were washed with PBS-T and incubated with peroxidase-conjugated secondary antibody diluted in PBS-T at room temperature for 1 hour. Membranes were washed in PBS-T and bound antibody was detected by enhanced chemiluminescence system Western Blotting Detection Reagents (Amersham Biosciences, Buckinghamshire, UK). Forty-eight hours after transfection, Western blot analysis was performed as above. The primary antibodies were mouse anti-human cyclin D1 monoclonal antibody (Santa Cruz, USA), rabbit anti-human MEKK2 polyclonal antibody (Santa Cruz), mouse anti-human p-Rb monoclonal antibody (Santa Cruz), mouse anti-human p-JNK monoclonal antibody (Santa Cruz) and mouse anti-human β-actin (Sigma). All experiments were repeated at least three times.

### Tumorigenicity in nude mice

All experimental procedures involving animals were in accordance with the *Guide for the Care and Use of Laboratory Animals* (NIH publications Nos. 80-23, revised 1996) and were performed according to the institutional ethical guidelines for animal experiment. BALB/c nude mice were employed for tumorigenicity analysis. The tumorigenicity of the stably pcDNA3-520b- or pcDNA3-transfected HepG2 cells was measured as follows. Forty-eight hours after transfection of pcDNA3-520b, the medium was replaced with fresh medium containing G418 to kill untransfected cells. HepG2 cells were transfected with NC or siRNAs targeting MEKK2 or cyclin D1. Forty-eight hours after transfection, cells were harvested by trypsinization, washed twice with sterile PBS, and resuspended at a concentration of 1×10^7^ cells/ml. Aliquots of 0.1–0.2 ml were injected subcutaneously into six 4- to 6-week-old Balb/c athymic nude mice. Tumor growth was measured after 3 days from injection and then every 3 days. Tumor volume (V) was monitored by measuring the length (L) and width (W) with calipers and calculated with the formula (L×W^2^)×0.5. Photographs were taken 4 weeks after the injection.

### Statistical analysis

All values are presented as means ± S.E.M. Each experiment was repeated at least 3 times. Statistical analysis was performed by the Student's *t* test. *P*<0.05 was considered significant. All statistical analysis was performed using SPSS13.0 software (Chicago, IL).

## Supporting Information

Figure S1 **MiR-520b inhibits expression of MEKK2 and cyclin D1 in tumors from mice in** **figure 3** **. The expression levels of MEKK2 and cyclin D1 in excised tumor tissues from mice were examined by western blot analysis.**
(TIF)

Figure S2 **MiR-520b decreases the levels of p-JNK and siMEKK2 abolishes levels of p-JNK in hepatoma cells.** The effect of miR-520b, siMEKK2-1 on phosphorylation levels of JNK (p-JNK) in HepG2 and H7402 cells was examined by western blot analysis. β-actin was used as an internal control.
(TIF)

Table S1 **The characteristics of clinical HCC patients.**
(DOC)

Table S2 **Sequences of DNA and RNA oligonucleotides.**
(DOC)
