# Supplementary figures and images for: MicroRNA-520b Inhibits Growth of Hepatoma Cells by Targeting MEKK2 and Cyclin D1
Source: PLoS One. 2012 Feb 3;7(2):e31450. doi: 10.1371/journal.pone.0031450 (PMC3272016; doi:10.1371/journal.pone.0031450)

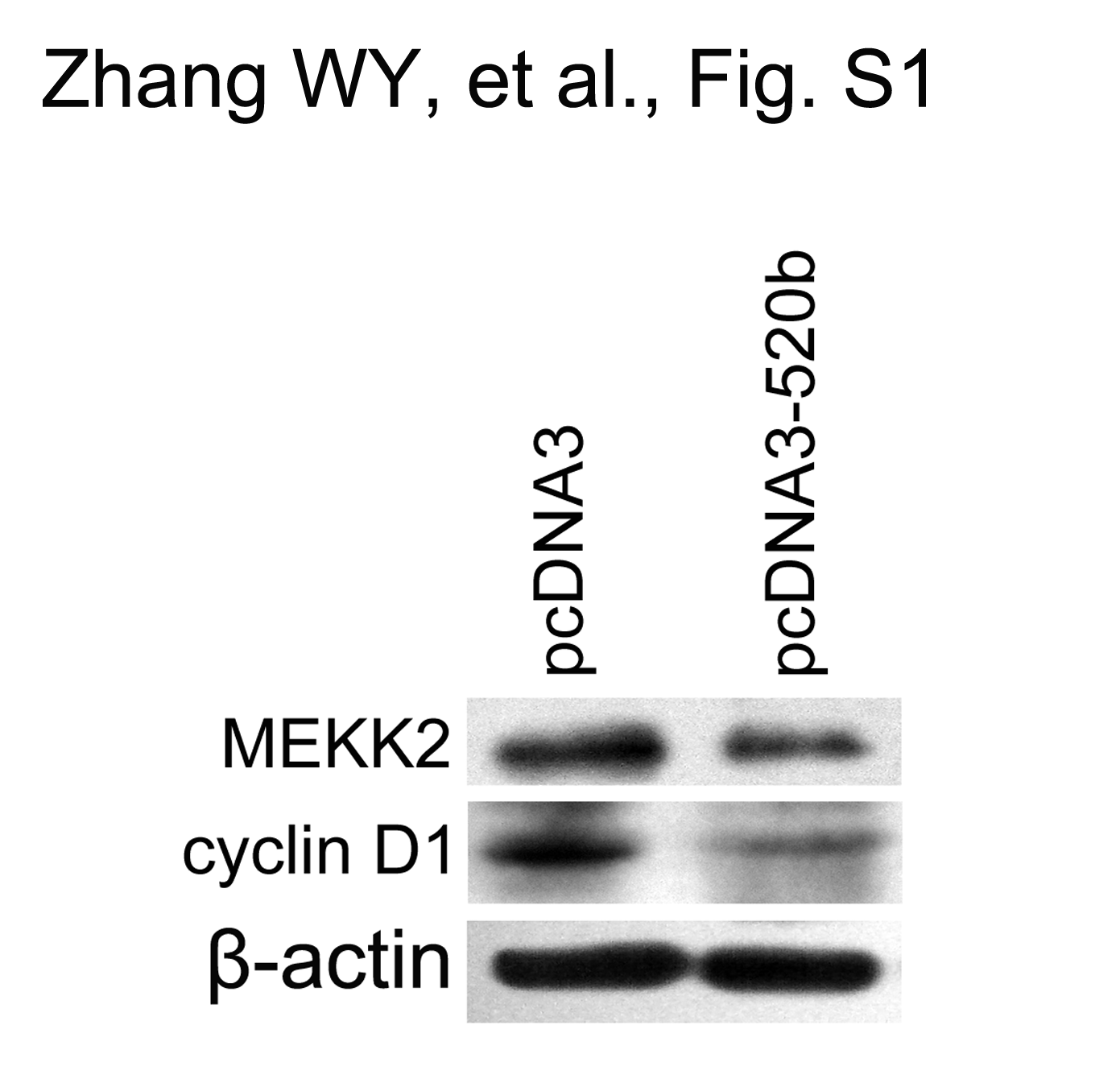

Supplement: Figure S1 — MiR-520b inhibits expression of MEKK2 and cyclin D1 in tumors from mice in figure 3 . The expression levels of MEKK2 and cyclin D1 in excised tumor tissues from mice were examined by western blot analysis. (TIF) [file pone.0031450.s001.tif]

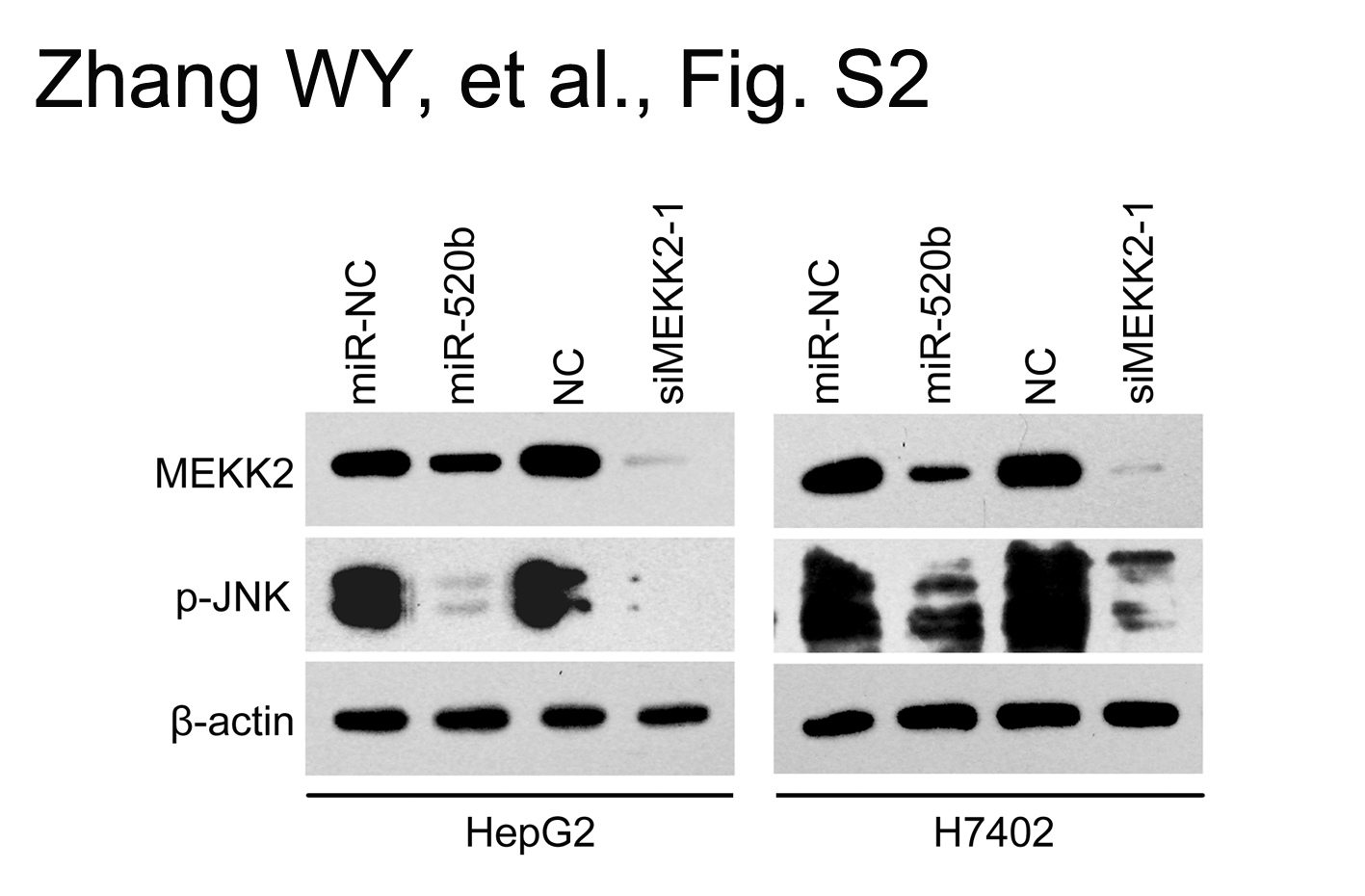

Supplement: Figure S2 — MiR-520b decreases the levels of p-JNK and siMEKK2 abolishes levels of p-JNK in hepatoma cells. The effect of miR-520b, siMEKK2-1 on phosphorylation levels of JNK (p-JNK) in HepG2 and H7402 cells was examined by western blot analysis. β-actin was used as an internal control. (TIF) [file pone.0031450.s002.tif]
