# Supplementary material for: MicroRNA-520b Inhibits Growth of Hepatoma Cells by Targeting MEKK2 and Cyclin D1
Source: PLoS One. 2012 Feb 3;7(2):e31450. doi: 10.1371/journal.pone.0031450 (PMC3272016; doi:10.1371/journal.pone.0031450)
Supplement: Table S1 — The characteristics of clinical HCC patients. (DOC) [file pone.0031450.s003.doc]

**Table S1.** The characteristics of clinical HCC patients

| Case No. | Age (yr) | Gender | Edmondson Grade | HBsAg |
| --- | --- | --- | --- | --- |
| 1 | 38 | M | III | + |
| 2 | 59 | M | III | + |
| 3 | 54 | M | III | + |
| 4 | 31 | M | III | + |
| 5 | 42 | F | II | + |
| 6 | 46 | M | II | + |
| 7 | 60 | M | II-III | + |
| 8 | 59 | M | III | + |
| 9 | 57 | F | II-III | + |
| 10 | 51 | M | II-III | + |
| 11 | 38 | M | II | + |
