# Supplementary material for: MicroRNA-520b Inhibits Growth of Hepatoma Cells by Targeting MEKK2 and Cyclin D1
Source: PLoS One. 2012 Feb 3;7(2):e31450. doi: 10.1371/journal.pone.0031450 (PMC3272016; doi:10.1371/journal.pone.0031450)
Supplement: Table S2 — Sequences of DNA and RNA oligonucleotides. (DOC) [file pone.0031450.s004.doc]

**Table S2.** Sequences of DNA and RNA oligonucleotides

| **Name** | **Sense Strand/Sense Primer (5′-3′)** | **Antisense Strand/Antisense Primer (5′-3′)** |
| --- | --- | --- |
| **MiRNA duplexes** | |  |
| miR-520b | AAAGUGCUUCCUUUUAGAGGG | CCCUCUACAGGGAAGCGCUUU |
| miR-NC | CGGUGAUAAUCUUUGUAGACG | CGUCUACAAAGAUUAUCACCG |
| **Primers for Cloning** | |  |
| pcDNA3-520b | CCGCTCGAGAAACAGAACCCCACCATCA | CCGGAATTCAACAGGGCAAATAAATGCA |
| pcDNA3-cyclin D1 | CCGGAATTCATGGAACACCAGCTCCTGTG | CCGCTCGAGTCAGATGTCCACGTCCCGCA |
| Cyclin D1 3′UTR-WT | CGTTCTAGACCACTCCTACGATACGCTAC | GGGGGCCGGCCGAACTTATCATCCTGGCAAT |
| Cyclin D1 3′UTR-Mut | CCATTCCATTTCCACTTTCAGTCCAATA | TATTGGACTGAAAGTGGAAATGGAATGG |
| MEKK2 3'UTR-WT | CGTTCTAGAAAAGACAAGAGGGAAAGT | GGGGGCCGGCCTGTCTTCCCATCGTCA |
| MEKK2 3'UTR-Mut | GTTCAGAGAGATAACTTTAGTACATAGT | ACTATGTACTAAAGTTATCTCTCTGAAC |
| **Primers for qRT-PCR** | |  |
| miR-520b | AAAGTGCTTCCTTTTAGAGGG | GCGAGCACAGAATTAATACGAC |
| U6 | AGAGCCTGTGGTGTCCG | CATCTTCAAAGCACTTCCCT |
